# Hybrid Nanomaterials: A Brief Overview of Versatile Solutions for Sensor Technology in Healthcare and Environmental Applications

**DOI:** 10.3390/bios14020067

**Published:** 2024-01-27

**Authors:** Norica-Carmen Godja, Florentina-Daniela Munteanu

**Affiliations:** Faculty of Food Engineering, Tourism and Environmental Protection, “Aurel Vlaicu” University of Arad, 2–4 E. Drăgoi Str., 310330 Arad, Romania; godja@attophotonics.com

**Keywords:** nanomaterials, sensors, environmental contaminants

## Abstract

The integration of nanomaterials into sensor technologies not only poses challenges but also opens up promising prospects for future research. These challenges include assessing the toxicity of nanomaterials, scalability issues, and the seamless integration of these materials into existing infrastructures. Future development opportunities lie in creating multifunctional nanocomposites and environmentally friendly nanomaterials. Crucial to this process is collaboration between universities, industry, and regulatory authorities to establish standardization in this evolving field. Our perspective favours using screen-printed sensors that employ nanocomposites with high electrochemical conductivity. This approach not only offers cost-effective production methods but also allows for customizable designs. Furthermore, incorporating hybrids based on carbon-based nanomaterials and functionalized Mxene significantly enhances sensor performance. These high electrochemical conductivity sensors are portable, rapid, and well-suited for on-site environmental monitoring, seamlessly aligning with Internet of Things (IoT) platforms for developing intelligent systems. Simultaneously, advances in electrochemical sensor technology are actively working to elevate sensitivity through integrating nanotechnology, miniaturization, and innovative electrode designs. This comprehensive approach aims to unlock the full potential of sensor technologies, catering to diverse applications ranging from healthcare to environmental monitoring. This review aims to summarise the latest trends in using hybrid nanomaterial-based sensors, explicitly focusing on their application in detecting environmental contaminants.

## 1. Introduction

In the wake of industrialization and globalization, a surge in toxic chemical emissions has permeated the global and local environment, escalating concerns over environmental pollution [1,2,3]. Prolonged exposure to these pollutants poses a substantial risk to human health [4,5], necessitating stringent ecological regulations and advancements in healthcare [6,7]. In the context of environmental pollution, there is a growing need for effective monitoring and detection of pollutants to mitigate their adverse effects on human health and the environment.

Nanomaterials [8,9] and nanotechnology [10,11] have emerged as transformative players in sensor development, offering a spectrum of advantages, including heightened sensitivity, selectivity, miniaturization, and real-time monitoring capabilities [12,13]. These advancements transcend disciplinary boundaries, leaving an indelible mark on environmental monitoring and industrial applications. Electrochemical sensors are favoured for monitoring water and air pollutants due to their cost effectiveness, rapid detection capabilities, high sensitivity, and selectivity.

**Figure 1:**
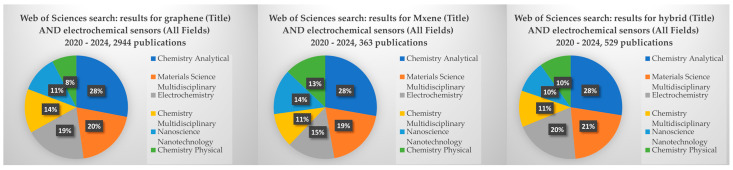
Web of Sciences search for “hybrid nanomaterials”, “MXene”, “graphene” and “electrochemical sensors”.

A search on the Web of Science research database using “hybrid nanomaterials” and “electrochemical sensors” within 2020–2024 yielded an extensive corpus of over 500 publications. Upon delving deeper into graphene and Mxene as specific materials, it became evident that a conspicuous bias towards graphene exists. Remarkably, there were 2879 publications dedicated to graphene in the context of electrochemical sensors, overshadowing the 351 publications focused on Mxene for the same application. This analytical insight underscores the intricate and multifaceted intersection between analytical chemistry and materials sciences, as illustrated in Figure 1.

The adaptability of electrochemical sensors, their ability to provide real-time measurements, and the incorporation of nanomaterials for enhanced performance make them valuable tools in environmental monitoring applications.

**Figure 2:**
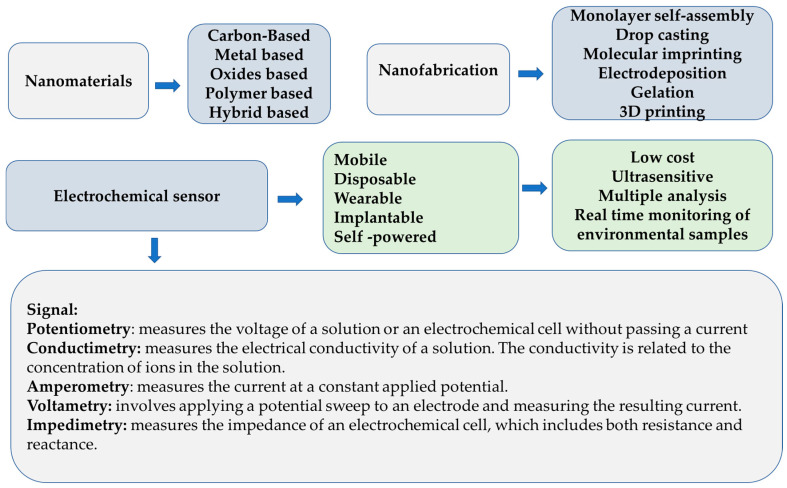
Diverse nanomaterials and fabrication techniques and varied signal detection methods contribute to developing highly sensitive and selective electrochemical sensors for various applications.

Figure 2 presents the recent trends in developing highly sensitive and selective electrochemical sensors that can be used for the detection of various analytes of interest.

Nanomaterials, serving as the building blocks at the nanoscale, and nanotechnology, involving the design and application of structures and devices at this scale, jointly offer new possibilities across diverse scientific and technological fields. They include applications in electrochemical sensors and biosensors [14], environmental analyses [15], and the fast and accurate detection of toxic substances, contributing to improvements in human life quality, life expectancy, and environmental protection [16]. Additionally, they find use in environmental monitoring [17], biosensors for the removal of toxic contaminants from drinking water [18], fluorescent biosensors for monitoring environmental pollutants [19], and the development of nanosensors and biosensors as a technique for air pollution detection in the foreseeable future [20]. Nanostructure-modified electrodes are also applied for the electrochemical detection of contaminants of emerging concern [21].

**Figure 3:**
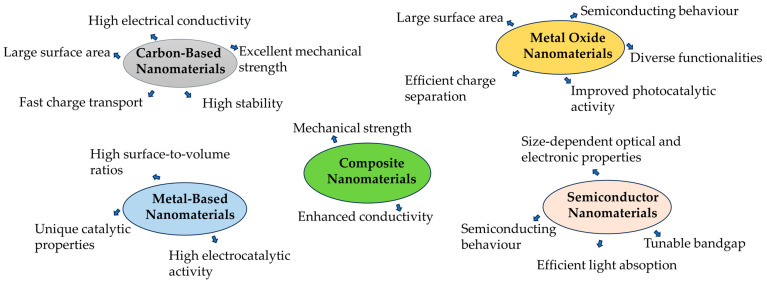
Properties of some nanomaterials.

Nanomaterials have ushered in a revolutionary era in sensor applications, harnessing their distinctive attributes, such as a high surface-to-volume ratio [22], adjustable optical, electrical, and magnetic characteristics, as well as heightened sensitivity and selectivity (Figure 3). Recent advancements in electrochemical sensor platforms are increasingly centred around the utilization of diverse nanomaterials, including MXene [23], carbon nanomaterials, metal oxide nanomaterials [15,24,25], metal nanoparticles [22], biomaterials [14], polymers [26], and hybrid materials [27]. These nanomaterials play a pivotal role in significantly enhancing the sensitivity and performance of electrochemical sensors across a wide array of domains, spanning environmental monitoring to healthcare applications. Their unique properties position them as invaluable tools for precise and efficient analysis, thus contributing substantially to various scientific and technological advancements.

In tandem with this progress, advanced nanofabrication techniques contribute to the creation of electrodes with enhanced sensitivity, employing methods such as monolayer self-assembly, drop casting, molecular imprinting, electrodeposition, in situ polymerization, hydrogenation, and 3D printing [28]. These advancements not only substantially improve environmental monitoring but also enhance accuracy and efficiency in analysis, as emphasized in a study by Barhoum et al. [29].

Furthermore, nanomaterials find versatile applications in the fabrication of biosensors for diverse purposes, including cancer detection [30], glucose sensing [31,32], and the development of optical biosensors for forensic analysis. This underscores the adaptability of nanomaterials in advancing sensor technologies across various domains, encompassing environmental monitoring to healthcare [33,34], forensic science [35], and the detection of trace antibiotics and various ecological contaminants [36].

## 2. Nanomaterials for Sensor Applications

**Figure 4:**
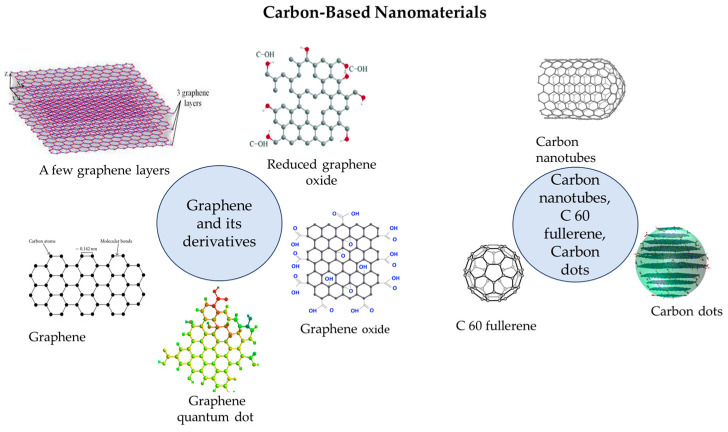
Graphene and its derivatives, carbon nanotubes, C60 fullerene, carbon dots.

The carbon-based nanomaterial family, including carbon nanotubes (CNTs), carbon dots (CDs), and fullerene (C60) (Figure 4), has garnered significant attention due to its low cost, easy preparation, biocompatibility, and notable electrochemical and optical properties. In the realm of carbon-based biosensors, the high conductivity, surface modification ease, and chiral structure of these materials have enhanced the performance of electrochemical and optical biosensors [30]. Nanocarbon materials, particularly allotropes like graphene and its derivatives, stand out as highly distinctive and promising substances in the scientific community. The functionalization of these materials remains a crucial technique to achieve enhanced performance [31]. The extraordinary characteristics of these naturally carbonaceous materials, including abundance, environmentally benign nature, high aspect ratio, impressive mechanical strength, optical behaviours, high chemical stability, low density, ease of handling and modification, excellent thermal and electrical conductivity, as well as porosity, have led to their widespread utilization in diverse areas across scientific and technological applications [32,33].

Carbon nanotubes (CNTs), graphene, and graphene oxide (GO) are widely utilized in sensors due to their exceptional electrical, thermal, and mechanical properties. CNTs and graphene-based sensors have been employed in various fields, including gas sensing, biosensing [34], and environmental monitoring [30].

Recently, MXenes have gained significant attention across various scientific fields due to their remarkable properties. These properties include excellent hydrophilicity, metal-like conductivity, abundant surface functional groups, a unique layered structure, a large specific surface area, and notable biocompatibility [23]. These features make MXenes an up-and-coming and versatile class of materials with potential applications in a wide range of scientific and technological domains, such as glucose biosensors [35,36], cytokines detection [37], for next-generation ultrasensitive sensors for the Internet of Things [22], for fabricating high-performance VOC-sensing devices [38].

Metal nanoparticles, including gold (Au), silver (Ag), and platinum (Pt), play a crucial role in sensing applications due to their unique optical, catalytic, and electrical properties. These nanoparticles are extensively utilized in optical sensing, electrochemical sensing, and surface-enhanced Raman spectroscopy (SERS)-based detection. Gold and silver nanoparticles [39,40], in particular, exhibit distinct optical and electrical properties attributed to their plasmonic behaviour. They enhance signal transduction in biosensors by amplifying signals generated from biological recognition events.

Magnetic nanoparticles, such as iron oxide (Fe_3_O_4_), are used in magnetic-based sensors [41]. Their magnetic properties can be utilized for the separation and manipulation of analytes. They can be functionalized with biomolecules to bind to target analytes, allowing for efficient capture and detection. These sensors can detect changes in magnetic properties caused by the presence of target analytes. Magnetic nanomaterials find applications in biosensors, drug delivery systems, and magnetic resonance imaging (MRI).

Semiconductor nanomaterials, like quantum dots (QDs) and metal oxide nanoparticles (e.g., zinc oxide, titanium dioxide), are widely employed in optical and electrochemical sensors. QDs offer size-tunable emission and excellent photostability, making them suitable for fluorescent-based sensing [42,43,44]. Metal oxide nanoparticles are commonly used for gas sensing and environmental monitoring. Titanium dioxide (TiO_2_) nanomaterials are extensively studied for their exceptional gas adsorption properties, making them valuable for detecting environmentally volatile organic compounds (VOCs) [25]. TiO_2_ has gained significant attention as a versatile semiconductor due to its high surface area, chemical stability, and unique electronic and optical properties. TiO_2_-based sensors exhibit high sensitivity and selectivity in detecting various VOCs, including benzene, toluene, xylene, and formaldehyde, and have been applied to identify air pollutants like nitrogen dioxide and ozone. Furthermore, TiO_2_ photocatalysis shows promise for degrading organic pollutants in air and water, highlighting its potential for environmental remediation [25].

On the other hand, two-dimensional (2D) materials, including molybdenum disulphide (MoS_2_), tungsten disulphide (WS_2_), and black phosphorus (BP) have gained prominence as auspicious materials for sensor applications. The ultrathin structure of these materials, coupled with their distinctive electronic properties, facilitates high-performance sensing across various domains. Specifically, these 2D materials demonstrate efficacy in sensing gases [45], chemicals, and biomolecules, showcasing their potential for advanced and versatile sensor technologies.

Nanocellulose, a notable nanomaterial within carbohydrate polymers, exhibits remarkable mechanical properties, biodegradability, and facile chemical modification. Despite these favourable characteristics, its practical applications have been hindered by limitations in functionalization. A study by Silva et al. [46] showed that screen-printed carbon electrodes on nanocellulose offer a solution that enables the detection of biologically relevant target species, including toxic metals, such as cadmium and lead, in artificial sweat.

A synergistic approach is achieved by integrating nanocellulose with metal–organic frameworks MOFs, which possess a large surface area, high porosity, and adjustable structure. This collaboration showcases the potential of nanocellulose-MOF composites for advanced and versatile sensor technologies and positions them as intriguing materials for multifunctional applications across various fields [47].

The synergy achieved by combining metal nanoparticles with carbon-based materials or metal oxides can enhance sensitivity, selectivity, and stability in sensing applications [48]. Hybrid organic–inorganic nanomaterials, which amalgamate organic and inorganic components, further exemplify this principle to achieve synergistic properties. A case in point is the combination of silica-coated quantum dots, which impart enhanced stability and biocompatibility, rendering them well-suited for applications in biological sensing [44].

The field of hybrid functional nanomaterials-based biosensors continues to evolve, with ongoing research focused on improving sensitivity, stability, biocompatibility, and integration with electronic devices. However, there are still some challenges that researchers are actively working to address. A few key areas where gaps exist in sensing activities are stability issues, such as aggregation, degradation, or loss of functionality over time, integration with complex, samples scalability and manufacturability [49].

## 3. Use of Hybrid Nanomaterials in Sensors for the Environmental Applications

### 3.1. Nanomaterials with Applications in Environmental Sensors

The combined effects of industrialization and globalization, both at international and local levels, have led to the significant release of hazardous chemicals into the environment [50]. Notable pollutants include carbon dioxide (CO_2_), sulfur oxides (SO_x_), nitrogen oxides (NO_x_), particulate matter (PM10), non-methane volatile organic compounds (NMVOCs), and heavy metals, such as cadmium (Cd), lead (Pb), and mercury (Hg).

Air pollutants can be categorized into two main groups. First, primary gases are those produced either naturally or by human activities, encompassing substances like carbon monoxide (CO), nitrogen dioxide (NO_2_), ammonia (NH_3_), sulfur dioxide (SO_2_), and nitrogen monoxide (NO). Second, secondary gases are formed through the interaction of pollutants from the first group, including ozone (O_3_), sulfur trioxide (SO_3_), ammonium (NH_4_), and particulate matter [51].

Industrial wastewater, a by-product of various manufacturing processes and factory instrument cleaning, poses a significant environmental threat by contributing to water pollution. It contains harmful substances, such as chemicals, heavy metals, and microorganisms, detrimental to aquatic life and ecosystems [52]. Water contamination, driven by the release of heavy metal ions from industrial activities, poses a persistent environmental challenge due to the nonbiodegradable nature of these contaminants, including chromium (Cr), arsenic (As), cadmium (Cd), mercury (Hg), and lead (Pb). These toxic ions adversely affect plants, animals, and humans, even at low concentrations.

Given these pollutants’ diverse and harmful nature, there is a pressing need to develop environmental pollutant sensor platforms. These platforms must exhibit sensitivity, reliability, and cost effectiveness. By achieving these characteristics, such sensor platforms can effectively monitor and detect the presence of pollutants in the air, providing valuable data for environmental management, regulatory compliance, and public health protection. The importance of these sensors extends beyond local boundaries, addressing the global impact of industrial activities on our environment and well-being [14].

Various nanomaterials, including metal nanoparticles (copper, iron, zinc, silver, gold, nickel), carbon-based nanomaterials (carbon nanotubes, carbon nanofibers, graphene, graphene oxide, fullerenes), and polymers (phenol-formaldehyde resin, resorcinol, poly-methyl methacrylate, chitosan, polyvinyl alcohol, poly(acrylic acid)), serve as electrode materials for detecting heavy metal ions (Table 1).

The advancement of sensor technologies involves using engineered nanoparticles, such as magnetite [53] and nanocomposites like Ce^3+^-doped CuO. For instance, magnetite nanoparticles have shown promise in water pollution control and detection. Additionally, nanocomposites like Ce^3+^-doped CuO have been employed to detect various contaminants in water, highlighting the diverse applications of nanomaterials in water quality monitoring [16].

Integrating multiple nanomaterials allows for synergistic effects, where combining different properties, such as high surface area and electrical conductivity, catalytic activity, optical properties, and biocompatibility, leads to improved sensor performance. For example, metal–organic framework (MOF) materials exhibit superior characteristics, enhancing thermal and mechanical stability, creating interconnected pores, improving conductivity, and boosting chemical and electrochemical properties [54]. Nanomaterials based on a three-dimensional (3D) polymer–metal–carbon framework are used to remove chemical and biological contaminants efficiently [55]. MOFs are widely used in catalysis, adsorption, separation, and energy storage. As catalysts in electrochemical sensors, the authors in [56] show that the design, including metal cations, synthetic ligands, and structure, is critical for improved applications in environmental monitoring, food safety control, and clinical diagnosis.

Hybrid materials are particularly useful in sensor preparations as their properties can be easily adjusted by changing the proportion of one component. This flexibility allows for customization of sensor performance, making them suitable for a range of detection methods, such as electrochemical [57], calorimetric, and fluorescence sensors [54].

In a critical review work by Gaviria-Arroyave et al. [19] on fluorescence biosensors based on nanomaterials for environmental pollutant monitoring like heavy metals, pesticides, and so-called emerging contaminants, it was shown that nanomaterials, such as gold nanoparticles, nanoclusters, graphene (G), graphene oxide (GO), transition metal dialcogens (TMDC), carbon dots (CD), quantum dots (QD), and metal–organic frameworks (MOF) could be used for the development of fluorescence detection systems. By carefully designing and engineering the hybrid nanomaterials, biosensors can be tailored to specific applications and analytes [58].

Achieving reliable and reproducible results across different laboratories and manufacturing processes is challenging. Standardizing fabrication methods [59], protocols and characterization techniques are essential for ensuring consistent and reproducible performance of hybrid nanomaterials-based biosensors [60]. Environmental sensors based on hybrid materials have seen significant advancements in recent years. These sensors offer improved detection capabilities, increased sensitivity, and enhanced selectivity for monitoring various environmental parameters. For practical applications, it is crucial to develop hybrid biosensors that are cost-effective and scalable for mass production. Finding economically viable synthesis methods, optimizing manufacturing processes, and reducing the overall cost of the sensors are areas of ongoing research.

Here are a few examples of state-of-the-art ecological sensors based on hybrid materials.

Particulate matter (PM) sensors, which are crucial for monitoring air pollution [61,62], can detect analytes by measuring changes in optical properties, such as fluorescence, absorbance, or refractive index [63].

The authors in [64] made significant strides in synthesizing and utilizing carbon dots (CDs) as a highly sensitive and efficient fluorescence probe for detecting both soluble and insoluble Cr(VI) in ambient particulate matter samples. They demonstrate that the passivation of CDs’ surface with 4-pyridine carboxaldehyde and thiourea produced highly luminescent f-CDs with an impressive quantum yield of approximately 76%. When applied to field particulate matter samples, the fluorescence method achieved a method detection limit (MDL) of 0.32 ng/m^3^ for total Cr(VI) quantification.

Hybrid nanomaterials, such as metal oxides combined with carbon nanomaterials or metal nanoparticles, have been used to develop highly sensitive gas sensors [65]. These sensors can detect gases, such as carbon monoxide, nitrogen dioxide, volatile organic compounds (VOCs), and various environmental pollutants.

#### Environmental Monitoring

In the electrochemical detection of environmental contaminants of concern (CECs), researchers employ various nanomaterials, such as nanoparticles, nanowires, graphene, and nanotubes to modify electrodes, including Glassy Carbon Electrode (GCE), Gold Electrodes, and Diamond-like Carbon Electrodes, in a three-electrode setup (working electrodes, reference electrodes, and counter electrodes) [28,66]. Surface modifications on working electrodes enable selective detection of specific analytes in electrochemical sensing systems [21].

Hybrid nanocomposites, consisting of nanomaterials and biomolecules (e.g., enzymes, antibodies, or DNA strands), have been employed in biosensors for environmental monitoring. These biosensors can detect specific pollutants, toxins, or pathogens present in the environment. Combining nanomaterials and biomolecules provides high sensitivity, selectivity, and biocompatibility for accurate and rapid environmental analysis [67].

Hybrid nanomaterials, such as quantum dots combined with organic dyes or plasmonic nanoparticles, have been utilized in optical sensors for environmental monitoring [68]. These sensors can detect analytes by measuring changes in optical properties, such as fluorescence, absorbance, or refractive index.

Hybrid nanocomposite materials, such as rGO combined with metal nanoparticles or metal oxides, have been used for water quality monitoring [65,69]. These sensors can detect contaminants, like heavy metals, in water sources. Challenges in detecting heavy metal ions persist in achieving optimal performance in terms of speed, sensitivity, and the ability to identify heavy metal ions selectively.

Environmental sensors based on hybrid materials have seen significant advancements in recent years (Table 1), especially for the detection of air pollutants [12,29,30,70,71,72], particulate matter [62,73], VOCs [22,38,71], water pollutants [24,57,66,69,74,75,76,77,78,79], and industrial chemicals (bisphenol A [80], formaldehyde [22], acetone [81], pesticides, and heavy metal pollutants in water [82]).

These sensors offer improved detection capabilities, increased sensitivity, and enhanced selectivity for monitoring various environmental parameters. Ongoing research focuses on further enhancing these sensors’ sensitivity, selectivity, and stability and their integration into intelligent monitoring systems for real-time environmental data acquisition and analysis.

**Table 1:** Examples of nanomaterials and their applications in sensing environmental pollutants.

Nanomaterial	Application	References
Carbon nanotubes, carbon nanofibers, C 60 fullerene	electrode materials for detecting heavy metal ions	[83,84,85,86,87]
Carbon dots	fluorescent sensing (gas molecules, pH, ions, and biological analytes)	[42,88,89]
Graphene, graphene oxide	electrode materials for detecting heavy metal ions	[24,90,91]
Reduced graphene oxide	gas sensing, biosensing, environmental monitoring	[74,80,81,92,93,94]
Graphene quantum dot	electrochemical biosensing	[95]
Mxene, Functionalized Mxene	VOC sensing device, healthcare sensors, Internet of Things	[22,23,35,36,37,38,96]
Metal nanoparticles (MeNPs)	copper, iron, zinc, silver, gold, nickel—electrode materials for detecting heavy metal ions; Au, Ag, Pt-optical sensing, electrochemical sensing	[66,79,97]
Magnetic nanoparticles + functionalization	biosensors, drug delivery, system MRI	[98]
Semiconductor nanomaterials	optical and electrochemical sensors	[42]
QD	Fluorescent-based sensing	[42]
Metal oxides-NPs	VOCs	[25]
2D materials like MoS_2_, WS_2_, black phosphorus	gas sensing, chemicals, biomolecules	[45]
Polymers: [99] phenol-formaldehyde resin, resorcinol, poly-methyl methacrylate, chitosan, polyvinylacohol, poly(acrylic acid)	electrode materials for detecting heavy metal ions	[66,78,100,101,102]
MOF, Nanocellulose-MOF	sensor technologies	[47]
Hybrid nanomaterials: carbon nanomaterials + MeNPs	PMs sensors	[62,73]
Me oxides + carbon nanomaterials or MeNPs	Gas sensors, VOCs, env. pollutants	[81,103]
Graphene oxide + Me NPs or Me oxides	water quality monitoring, heavy metals, org. pollutants, pathogens	[16,69]
Hybrid nanomaterials–Nanocomposites: Carbon nanomaterials + Me oxides NPs + polymer	VOCs detection	[104,105]
QDs + org. dyes or plasmonic NPs	optical sensors for environmental monitoring	[43,68,106]
Nanomaterials + biomolecules	biosensors for environmental monitoring	[17,107]

### 3.2. Nanomaterials in Sensor Technology

Integrating nanomaterials in sensor technologies presents challenges and promising avenues for future research [12,13]. The challenges include assessing the potential toxicity of nanomaterials and ensuring biocompatibility, especially for medical applications., scalability and costs, standardization, and long-term stability, seamlessly integrating nanomaterial-based sensors with existing technologies and infrastructure for practical implementation. Additionally, interference, reproducibility, and ethical considerations must be addressed.

Potential avenues for future development involve the creation of multifunctional nanocomposites, innovative nanomaterials responsive to environmental changes, and biodegradable options for sustainability. Advanced characterization techniques and machine learning integration offer opportunities for enhanced sensor functionality [108].

Collaboration between academia, industry, and regulatory bodies is crucial for standardization and accelerated development. Balancing these challenges and opportunities can unlock the full potential of nanomaterial-based sensors across various applications, from healthcare to environmental monitoring.

**Figure 5:**
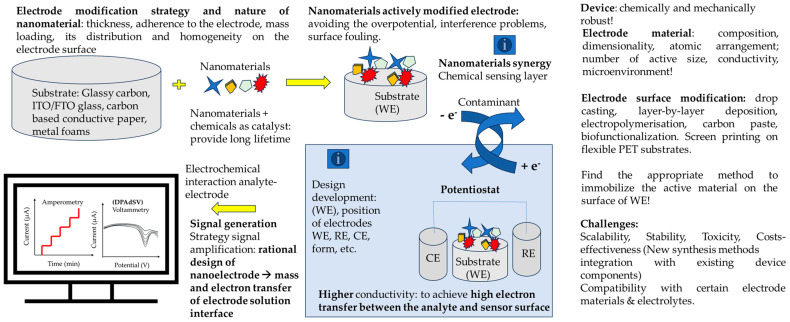
Integration of nanomaterials in electrochemical sensors.

In our opinion, screen-printed sensors based on nanocomposites with high electrochemical conductivity provide significant advantages for environmental sensing using electrochemical sensors (Figure 5). These sensors offer cost-effective production suitable for large-scale monitoring and allow for customizable designs that incorporate various nanocomposites, ensuring versatility. To enhance conductivity, incorporating hybrids based on carbon-based nanomaterials or graphene and its derivatives, functionalized Mxene appears to be the most effective solution.

Tapia et al. [85] developed a two-dimensional (2D) Sb-modified screen-printed carbon nanofiber electrode (2D Sbexf-SPCNFE) with the aim of improving the stripping voltammetric determination of Cd(II) and Pb(II). The electrode demonstrated excellent linear behaviour in the concentration range of 2.9 to 85.0 µg L^−1^ for Cd(II) and 0.3 to 82.0 µg L^−1^ for Pb(II) within an analysis solution of 0.01 mol L^−1^ HCl (pH = 2).

The authors of another study [76] introduce a new method for creating a cost-effective and easily fabricated amperometric sensor designed to detect low concentrations of NO^3−^ in real water samples. This approach involves printing a silver (Ag) working electrode and subsequently modifying it with electrodeposited copper (Cu) nanoclusters. The process was optimized to achieve a high catalytic activity for the electroreduction of NO^3−^, resulting in a sensitive sensor (19.578 μA/mM) with a low limit of detection (0.207 nM) and a dynamic linear concentration range of 0.05 to 5 mM or 31 to 310 mg/L. The sensors exhibited negligible interference effects from various analytes, and they were successfully applied to detect NO^3−^ in real water samples. Rubino and Queirós [109], in their review on the electrochemical determination of heavy metal ions using screen-printed electrode (SPE)-based sensors, focused on various metal-based SPEs, including bismuth-based, Antimony-based, and Gold-based SPEs. They also explored modified SPEs, such as those with Ag nanostructures, carbon nanofibers (CNFs/SPCE), Nafion, carbon-covered halloysite, and multi-walled carbon nanotubes (MWCNTs) (Nafion/C–Hal/MWCNTs modified SPCE). The review highlighted the challenges posed by interfering metal ions in real samples as a limitation for the application of SPEs. Despite this, the authors emphasized the significance of developing SPE-based devices with potential applications in various areas, foreseeing their pivotal role in the low-cost sensor market in the near future. In the study by Zhao et al. [110], the researchers modified the working electrodes of screen-printed carbon electrodes (SPCEs) with silver-gold bimetallic nanoparticles using electrochemical deposition. This modification aimed at enhancing the detection of chromium (VI). The results showed a linear range and limit of detection (LOD, identified by three times the signal-to-noise ratio) of 0.05–5 ppm and 0.1 ppb for Cr(VI), and 0.05–1 ppm and 0.1 ppb for Cr(III), respectively. The use of gold-silver nanoparticles on the electrochemical sensor array allowed for the simultaneous determination of chromium (III) and (VI) in wastewater samples.

Another study reports the results of experiments using screen-printed electrodes for potential applications as portable sensors or for on-field detection of Hg(II) and Cr(VI). The results suggest that the proposed sensor has the potential to pave the way for the development of robust and high-performance multiplex sensing approaches for metal ion detection. The multiplex electrochemical sensor was created by covalently functionalizing graphene oxide with thymine and carbohydrazide (Thymine-GO-Carbohydrazide, T-GO-C) through an epoxide ring cleavage, employing a simultaneous reduction approach. With its large surface area, good conductivity, and functionalization, the sensor exhibited enhanced selectivity for Hg(II) and Cr(VI) compared to other metal ions. The developed multiplex electrochemical sensor demonstrated a linear range for Hg(II) and Cr(VI) concentrations above 5 ppb, with minimum detection limits estimated at 1 ppb for Hg(II) and 20 ppb for Cr(VI).

The review by A. O. Idris et al. [111] focuses on monitoring selected organic contaminants in water utilizing an electrochemistry technique due to intrinsic benefits, such as simplicity, portability, cost, and improved sensitivity. The authors recommend using screen-printed electrodes for their design that prevent modifier leaching into analytical solutions, ensuring effective interaction with analyte solutions for reliable analysis of real-world samples.

With their high electrochemical conductivity, these sensors exhibit enhanced performance. They are portable, often miniaturized, and well-suited for on-site applications. Their rapid response times make them ideal for real-time monitoring, and their compatibility with IoT platforms facilitates data collection and analysis for smart environmental monitoring systems. The optimal choice of sensor depends on specific application requirements, such as target analytes, sensitivity, detection limits, and environmental conditions, leading researchers to optimize configurations accordingly.

**Figure 6:**
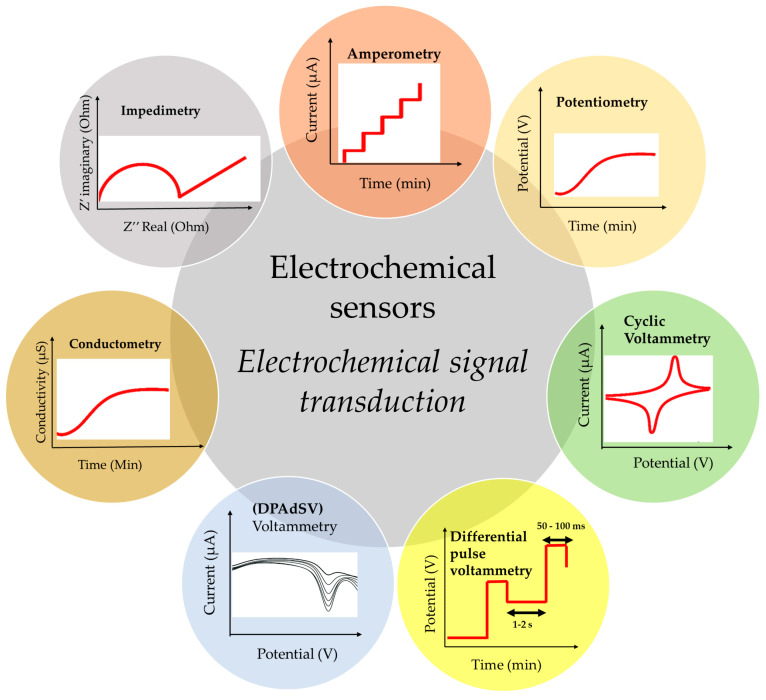
Electrochemical signal transduction–electrochemical sensors.

In the pursuit of identifying and quantifying water and air pollutants, various sensors come into play (refer to Table 2). Notably, electrochemical sensors have seen significant development in recent years, particularly for the detection of heavy metals. Figure 6 provides a schematic representation of the detection methods employed by these sensors. This literature review highlights the prevalence of voltametric detection methods, with a focus on techniques such as differential pulse adsorptive stripping voltammetry (DPAdSV), differential pulse voltammetry (DPV), direct linear sweep voltammetry (LSV), and amperometry for the detection of heavy metals in wastewater (as detailed in Table 3). These electrochemical methods offer effective means of identifying and quantifying heavy metal pollutants.

Alternative sensor types come into play for the detection of organic substances, volatile organic compounds (VOCs), and industrial gases. Optical sensors, gas sensors, and biosensors are viable options for such applications.

Sensors for pollutant detection can be categorized based on working principles, detection methods, and targeted pollutants. Working principles include chemical sensors (gas sensors, ion-selective electrodes), physical sensors (optical, thermal, acoustic), and biological sensors (enzymatic, immunosensors, DNA sensors). Detection methods encompass electrochemical, optical, and mass-based sensors. Regarding types of pollutants, sensors are designed for air quality (particulate matter, gases), water quality (pH, dissolved oxygen), and soil quality (nutrients, pH) and can be organized as stationary, mobile, or part of remote sensing systems.

To obtain high-sensitivity/high-stability electrochemical enzymatic biosensors for detecting phenolic pollutants and the strategy for the product transfer of portable analytical devices, effective immobilization of enzymes and nanomaterials has been extensively researched in recent years. Zhang et al. [115] provided an overview of recent progress toward electrochemical enzymatic biosensors, including the features, mechanisms, and traditional enzyme immobilization methods. The authors show that there is no one-size-fits-all method of enzymatic immobilization.

An optical sensor array based on gold nanoparticles functionalized by mercaptoundecanoic acid, 2-mercaptoethanesulfonate, and a 1:1 mixture of the two ligands was used for the recognition and quantification of seven toxic metals (detection limit arsenic −10 µM, barium −5 µM cadmium 15 µM, cerium 5 µM, chromium 5 µM, lead 5 µM, and mercury 8 µM) [97].

On the other hand, the detection speed is crucial for real-time monitoring and wearable biosensors [116]. In this case, electrochemical biosensors may have an advantage over chemiluminescence-based biosensors, as they can provide faster acquisition times. However, the competition between photons and electrons is still an open question and ultimately depends on the specific requirements of the detection system [117].

Terahertz (THz) sensing technology has attracted tremendous interest in recent decades due to its unique applications in various fields, including wireless communications, spectroscopy, imaging, and non-invasive detection. The development of metamaterials and two-dimensional (2D) materials has further spurred advances in THz biosensing due to their unusual optical and electrical properties. There are significant advances in THz biosensing based on artificial electromagnetic subwavelength structure and the potential for these biosensors to revolutionize non-invasive detection in various fields, including healthcare, security, and environmental monitoring [118].

Sensors based on graphene and its derivatives have garnered attention for pollutant detection, exploiting graphene’s unique properties, such as excellent electrical conductivity, a large surface area, and exceptional chemical reactivity. These results have led to the development of diverse graphene-based sensors for detecting pollutants in various environmental matrices. Numerous studies highlight applications in pollution monitoring, utilizing carbon nanomaterials, highly porous metal–organic framework (MOF), pure, mixed, and doped metal oxides (MOX), electrospun nanofibers, graphene oxide (GO) and reduced graphene oxide (rGO) in gas sensors [119] for water and air pollution control. Functionalization of graphene enhances selectivity and sensitivity to specific pollutants. These sensing platforms are widely employed for monitoring environmental factors, relying on resistance change as a sensing mechanism, with applications in temperature, humidity, and volatile organic compounds (VOCs). Moreover, graphene-based sensors are utilized in electrochemical sensors for detecting heavy metal ions in water, offering advantages in sensitivity, selectivity, and rapid response. Despite promising advancements, challenges [90], such as reproducibility, stability, and scalability, persist.

Ongoing research, as highlighted by [120], aims to address challenges and optimize graphene-based sensors for real-world applications in environmental pollutant detection. The literature emphasizes the continuous exploration and development of graphene-based sensing platforms, showcasing their potential impact on environmental monitoring. However, several challenges persist, including the need for real-world performance assessment and considerations regarding the impact of environmental contaminants. Contamination poses practical hurdles, underscoring the importance of obtaining uncontaminated samples. Adopting lower-grade graphene is feasible, but widespread usage relies on the availability of high-quality graphene and associated costs. The anticipated integration of wireless technology into graphene sensors could enhance their utility in the Internet of Things era. Combining graphene with organic polymers, biomolecules, and inorganic nanoparticles has shown promise in improving sensitivity, selectivity, repeatability, and expanding detection ranges. Over the past decade, graphene and its composites have witnessed significant advancements in controlled design. Various graphene-derived materials, such as reduced graphene oxide (rGO), 3D graphene, graphene quantum dots (GQDs), and doped graphene, remain attractive for nanocomposite synthesis and electrode modification.

#### Mxene Based Sensors

The study by Chen et al. [22] demonstrates that optimal Au functionalization of 2D MXene is an effective way for fabricating high-performance VOC-sensing devices.

The authors in [9] describe dynamic sensing experiments that demonstrate the enhanced performance of flexible sensors through the optimal decoration of Au nanoparticles (NPs) on Ti_3_C2Tx MXene. These sensors show elevated response and selectivity, particularly in detecting formaldehyde. The Au−Ti_3_C2Tx gas sensors exhibit an impressively low detection limit (92 ppb) for formaldehyde at room temperature. The sensors provide reliable gas response, low noise levels, ultrahigh signal-to-noise ratio, high selectivity, and the ability to detect formaldehyde at parts per billion levels. Theoretical elucidation through density functional theory simulations highlights the mechanism by which Au−Ti_3_C2Tx senses formaldehyde.

The results suggest that decorating noble-metal NPs on MXenes, such as Au−Ti_3_C2Tx, is a promising strategy for developing next-generation ultrasensitive sensors, particularly for Internet of Things (IoT) applications.

For detecting various oxygen- and hydrocarbon-based VOCs at room temperature using pure Ti3C2Tx and graphene as controls. Au-Ti3C2Tx hybrid materials were successfully synthesized [38] by a simple solution mixing method, which has excellent potential for scalable fabrication. It was shown that optimal Au functionalization of 2D MXene is an effective way to fabricate high-performance VOC sensors [22]. The research presents a stretchable and wearable conductometric VOC sensor based on microstructured MXene/polyurethane (PU) core-sheath fibres. The study successfully achieved a highly stretchable gas sensor that provides reliable electrical feedback while minimizing interference from external strain through microwrinkle engineering. The stretchable fibre sensor was integrated into the fabric, creating a wearable gas sensor with good gas permeability and excellent mechanical and sensing stability. To enhance the sensing performance of the MXene/PU fibre, microstructures, including microcracks and micro wrinkles, were incorporated into the fibre sheath. The microcracks were designed to amplify the swelling-induced resistance variation of the conductive sheath, resulting in an improved sensing response that was 40% higher compared to the flat core-sheath fibre. This innovative approach demonstrates the potential for creating flexible and stretchable gas sensors with enhanced sensitivity for wearable applications.

Sensors based on TiO_2_ have been developed for detecting various VOCs, including benzene, toluene, xylene, and formaldehyde, with high sensitivity and selectivity. TiO_2_-based sensors have also been used to detect gases, such as nitrogen dioxide and ozone, which are major air pollutants. In addition, TiO_2_ photocatalysis has been explored for the degradation of organic contaminants in water and air, making it a promising material for environmental remediation [25].

Nanofibers of ZnO-SnO_2_ nanocomposites doped with Au crystals were successfully synthesized using an electrospinning method to enhance H_2_S gas sensing performance. The fabrication involved spinning a mixed solution of zinc acetate dihydrate and tin (II) chloride dihydrate directly onto interdigital Pt electrodes, followed by thermal treatment to convert the nanofibers into ZnO-SnO_2_ nanocomposites with Au crystal doping. Gas sensitivity to H_2_S was notably improved, showing an approximately 700% enhancement with the optimal doping concentration of Au [103].

Ferrite-based sensors offer advantages, such as cost-effectiveness, rapid detection, and simple operational procedures, making them suitable for miniaturization into portable sensing tools for real-world applications. However, a significant limitation highlighted in the literature is the absence of real-time applications; as of now, all sensors have been evaluated only under laboratory conditions. Commercializing or translating to end-users faces challenges and requires satisfactory real-time validation at all levels before widespread implementation [121].

Electrochemical sensors based on MOF catalysts currently exhibit limitations in laboratory conditions [56]. Further research on MOF-based catalysts is necessary to enhance their electrochemical properties and broaden their applications. The ongoing development in nanoscience and biotechnology is expected to unlock more excellent prospects for MOF-based electrochemical sensors, particularly in environmental monitoring.

The study conducted by Xu et al. [69] focused on the adsorptive stripping voltammetry determination of hexavalent chromium (Cr(VI)) using a pyridine-functionalized gold nanoparticles/three-dimensional graphene electrode. The electrode was prepared through the electroreduction of graphene oxide, electrodeposition of gold nanoparticles, and self-assembly of pyridine groups. The resulting electrode exhibited high sensitivity, selectivity, and stability in detecting Cr(VI), making it a promising tool for accurately determining hexavalent chromium in various samples.

A novel material based on silver nanoparticles-2D biphenol-biphenoquinone nanoribbons was developed by using a fast and straightforward redox reaction between Ag+ ions and BP molecules, followed by the generation of a hydrogen bonding network between prepared BPQ and BP molecules at reduced solution pH [79]. This material was utilized to modify a graphite paste electrode, exhibiting a low detection limit of 2.0 × 10^−12^, a wide linear range, good selectivity, and sensitivity for ultra-trace Cr(VI) determination. The redox reaction between BP and Cr(VI) was investigated, and the product was characterized. The modified electrode was successfully applied to determine trace amounts of Cr(VI) in river water and electroplating wastewater, yielding results comparable to ICP-AES. The sensor demonstrated good recovery values for spiked Cr (VI) concentrations in tap water, river water, and electroplating wastewater, indicating its applicability for ultra-trace Cr (VI) determination in real samples.

In the study by Karthika et al. [77], a g-C_3_N_4_/AgM nanocomposite was prepared using a simple sonochemical method and employed as an excellent electrode material for Cr^6+^ reduction in electrochemical analysis. The amperometric i-t curve exhibited remarkable sensitivity (65.8 mAmM^−1^cm^−2^), wide linear ranges (0.1–0.7 μM), and an exceptionally low detection limit (0.0016 μM). The proposed g-C_3_N_4_/AgM modified electrode effectively detected Cr^6+^ in various water samples (tap water, drinking water, river water, and industrial water), achieving good recoveries. Moreover, the g-C_3_N_4_/AgM modified electrode demonstrated excellent selectivity, stability, reproducibility, and repeatability. These findings suggest the potential applicability of the proposed g-C_3_N_4_/AgM modified electrode for determining Cr^6+^ in real samples.

In a study by Li et al. [80], a voltammetric sensor was employed to detect bisphenol AP (BPAP) in industrial wastewater. NiCoMnO_4_-rGO nanocomposites were synthesized using a simple and convenient method, leading to the construction of voltammetric sensors. The peak current (Ipa) demonstrated a linear dependence on BPAP concentrations ranging from 0.005 μM to 7 μM under optimal conditions. The achieved detection limit was 2 nM (S/N = 3). The developed system was successfully utilized to determine BPAP in industrial wastewater, employing the standard addition method.

The discussed strategies for signal amplification in nanostructured electrochemical sensors [122] for environmental pollutants highlight the importance of electrode material parameters, including dimensionality, atomic arrangement, and composition, emphasizing mass transfer as a critical factor. The reproductivity challenge in complex detection environments can be addressed through controlled synthesis or design of nanomaterials for sensing elements and signal amplification, coupled with a deep understanding of surface or interface processes in recognition events. In the long term, 2D nanomaterials, mainly for constructing high-performance electrochemical sensors, are deemed crucial due to their ultrathin structure, confining electrons/holes to a plane and enhancing sensitivity. Efforts should also focus on in situ characterizations and a combined theory–experiment approach for electrode material design and understanding electrochemical processes. The studies investigating the effects of electrode material parameters, such as dimensionality, atomic arrangement and composition, on the mass and electron transfer of nanoelectrodes, highlight the insufficient attention paid to mass transfer as a critical factor. It emphasises the major challenge of ensuring the reproducibility of high performance in nanostructured electrochemical sensors, which can be addressed by controlled synthesis or design of nanomaterials and a deep understanding of surface or interfacial processes in detection processes.

Aptasensors are suggested as a smart methodology to address sensor interference in organic pollutant detection. Hydrophobic or anti-electrode passivation nanomaterials like polyoxometalates, chitosan, nickel oxide, or diamond can mitigate electrode fouling during organic pollutant detection. To prevent nanomaterial leaching, an electrodeposition method is advised for immobilizing nanomaterials on conducting substrates, and at least two electrochemical techniques should be employed in analyte detection. For industrial applications, the incorporation of sensors prepared via electrodeposition into water treatment reactors is recommended by authors for consistent and reproducible results in the electrochemical detection of organic pollutants.

The synthesis methodology and detection strategies for Cr(VI) in another study [78] provide a convenient and cost-effective approach for sensor fabrication applicable in various fields, such as environmental monitoring, biological studies, and domestic drinking water analysis. The sensor, developed through chemical oxidative and electrochemical polymerization of the monomer PP and its polymers (poly(PP)-O and poly(PP)-E), exhibited selective sensitivity to Cr(VI) with a calculated limit of detection (LOD) at 0.106 µM.

In a recent study [104], multi-walled carbon nanotubes (MWCNTs) were successfully functionalized with copper oxide nanoparticles (CuO NPs) through a two-step process involving carboxyl group introduction and hydrothermal synthesis. The resulting CuO-functionalized CNTs were dispersed in polyvinylidene fluoride (PVDF) to create a polymeric composite electrode for detecting toxic volatile organic compounds (VOCs). The nanocomposites exhibited excellent sensing capabilities for various VOCs, with distinct voltage responses observed over time, offering a cost-effective, high-performance solution.

Motaghedifard et al. [57] developed an electrochemical sensor for selective and sensitive detection of Cr(VI) pollution in wastewater via polyaniline/sulphated zirconium dioxide/multi-walled carbon nanotubes nanocomposite hybrid material. To address the poor electron transfer kinetics and selectivity issues in carbon-based electrochemical sensors, polyaniline (PANI) nanostructures were employed. However, an intermediate structure was necessary for the uniform dispersion of PANI nanostructures over carbon nanostructures. ZrO_2_ nanostructures, synthesized through coprecipitation and converted to sulphated nanostructures, served this purpose. The resulting PZrS nanocomposite-modified glassy carbon electrode (GCE) demonstrated a low detection limit of 64.3 nmol L^−1^ for Cr(VI) ions in industrial wastewater, exhibiting features such as a wide linear concentration range, high stability, and good reproducibility [57].

The detection methods (Figure 6) employed by sensors are critical for accurately identifying and quantifying pollutants in air and water.

Electrochemical sensors, such as those utilizing voltametric techniques like DPAdSV, DPV, LSV, and amperometry (Table 3), offer high sensitivity and specificity, particularly for detecting heavy metals in water. On the other hand, optical sensors, gas sensors, and biosensors contribute to a versatile approach for detecting organic substances, VOCs, and industrial gases. Electrochemical sensors are known for their selectivity, sensitivity, and applicability to trace-level detection, while optical sensors, gas sensors, and biosensors provide real-time data and cater to specific types of pollutants. Ongoing advancements in sensor technology, including miniaturization and smart sensor networks, contribute to more efficient and widespread environmental monitoring, allowing for a comprehensive understanding of pollution levels in air and water.

Looking ahead, the future trajectory for electrochemical sensors involves a concerted effort to elevate sensitivity levels. This pursuit entails exploring diverse strategies to enhance the accuracy and reliability of pollutant detection, particularly at lower concentrations. Integration of nanotechnology stands out as a promising avenue, leveraging materials like nanoparticles and nanotubes to capitalize on their high surface area and distinctive properties. Advanced materials, with superior conductivity and catalytic features, are being employed alongside surface modifications to tailor sensor responses, thereby augmenting both sensitivity and selectivity. Further strides in miniaturization and microfabrication are anticipated, streamlining sensor components, and paving the way for compact, portable devices. This not only facilitates deployment but also heightens sensitivity by reducing analyte diffusion distances. Simultaneously, improvements in signal processing algorithms and data analysis techniques are enhancing the interpretation of sensor signals, enabling meaningful insights from subtle electrochemical responses.

In our opinion, innovative electrode designs, including three-dimensional structures or porous materials, should be explored to optimize the electrochemical interface, fostering improved sensitivity through enhanced analyte interaction. Integration into the Internet of Things (IoT) is a notable trend, connecting electrochemical sensors to real-time data transmission and remote monitoring platforms. This not only strengthens overall environmental monitoring networks but also enables swift responses to emerging pollution events. Simultaneously, a focus on energy-efficient sensor technologies ensures continuous and prolonged monitoring without frequent maintenance, contributing to sustained sensitivity over extended periods.

## 4. Conclusions

In conclusion, the integration of nanomaterials in sensor technology presents both challenges and exciting prospects for future research. Challenges, including potential toxicity, biocompatibility, scalability, costs, standardization, and long-term stability, must be carefully addressed to ensure practical implementation. Additionally, considerations such as interference, reproducibility, and ethical aspects are crucial components of this ongoing exploration.

The possibilities for future development in nanomaterial-based sensors involve the creation of multifunctional nanocomposites, innovative nanomaterials responsive to environmental changes, and sustainable, biodegradable options. Advanced characterization techniques and the integration of machine learning provide opportunities to enhance sensor functionality. Collaboration between academia, industry, and regulatory bodies is deemed crucial for standardization and accelerated development.

Specifically, our perspective highlights the advantages of screen-printed sensors based on nanocomposites with high electrochemical conductivity, especially in environmental sensing using electrochemical sensors. These sensors offer cost-effective production suitable for large-scale monitoring, with customizable designs incorporating various nanocomposites, ensuring versatility. The incorporation of hybrids based on carbon-based nanomaterials or graphene and its derivatives, along with functionalized Mxene, emerges as an effective solution to enhance conductivity and overall sensor performance.

Notably, these high-electrochemical conductivity sensors exhibit enhanced performance characteristics, making them well-suited for on-site, real-time environmental monitoring applications. Their portability, miniaturization, rapid response times, and compatibility with IoT platforms contribute to their potential for smart environmental monitoring systems. The optimal choice of sensor depends on specific application requirements, emphasizing the need for researchers to tailor configurations accordingly.

In parallel, the discussion on detection methods emphasizes the critical role sensors play in accurately identifying and quantifying pollutants in air and water. Electrochemical sensors, with their selectivity and sensitivity, particularly in detecting heavy metals, complement the versatility of optical sensors, gas sensors, and biosensors. Ongoing advancements in sensor technology, ranging from miniaturization to smart sensor networks, contribute to more efficient and widespread environmental monitoring, providing a comprehensive understanding of pollution levels.

Looking forward, the future trajectory for electrochemical sensors involves a concerted effort to elevate sensitivity levels, exploring strategies such as nanotechnology integration, advanced materials, miniaturization, and innovative electrode designs. The integration of sensors into the Internet of Things (IoT) further enhances real-time data transmission and monitoring capabilities. Overall, this comprehensive approach aims to unlock the full potential of sensor technologies, ensuring their effectiveness in diverse applications, from healthcare to environmental monitoring.

## Figures and Tables

**Table 2:** Examples of air and water pollutants, analytes, and their detection methods.

Pollutants	Detection	
Air pollutants		
(CO), nitrogen dioxide (NO_2_), ammonia (NH_3_), sulfur dioxide (SO_2_), and nitrogen monoxide (NO) Second, secondary gases are formed through the interaction of pollutants from the first group, including ozone (O_3_), sulfur trioxide (SO_3_), ammonium (NH_4_) Particulate matter (PM10, PM2.5) Volatile organic compounds (VOCs)	Electrochemical sensors Detection: amperometry, potentiometry, conductometry, impedance spectroscopy. Semiconductor sensors Detection: changes in resistance, capacitance, or other electrical properties are monitored when the semiconductor material interacts with the target analyte. Gas resistance sensors Detection: the electrical resistance of the sensor material is measured, and changes in resistance due to gas interactions indicate the presence and concentration of the target gas. Optical sensors, resonant mass sensors. Semiconductor sensors Electrochemical sensors	[22,25,30,47,63,65,70,104,112]
Water pollutants		
Heavy metals (e.g., lead, cadmium, mercury, chromium (VI), vanadium) Organic substances (e.g., pesticides, industrial chemicals):	Electrochemical sensors: Differential pulse voltammetry (DPV), Differential pulse adsorptive stripping voltammetry (DPAdSV), Direct linear sweep voltammetry (LSV). Ion-selective electrode sensors Detection: The membrane potential changes in response to the concentration of the specific ion being measured. This change in potential is measured and used to determine the ion concentration. Optical spectroscopy. Detection: by measuring the absorption, emission, or scattering of light, optical spectroscopy allows for the identification and quantification of specific molecules or chemical compounds Biosensor-based sensors The choice of bioreceptor depends on the target analyte, and the transducer converts the biological response into an electrical, optical, or other measurable signals. Optical sensors utilize the interaction between light and matter to detect and quantify analytes. The optical properties of the sensing material change in response to the presence of the target analyte, and these changes are measured using various optical techniques.	[57,66,69,74,75,77,78,79,82,97,113,114]

**Table 3:** The techniques employed in the detection and quantification of different analytes, along with the use of hybrid nanomaterials in sensor fabrications, vary depending on the specific application and targeted analytes.

Electrode/Material/Modifier	Target Analyte	Linear Range	Sensitivity	Techniques	Limit of Detection (LOD)	References
rGO functionalized metal-doped SnO_2_ nanocomposites	Selective detection of Cd (II) and Cr (VI) ions	Cd: 0.1–50 ppb	(~1.4 µA/ppb (Co doping); ~2.6 µA/ppb (Fe doping)). 1 wt% dopping of Co and Fe into SnO_2_. ((3.2 µA/ppb) for Cd(II) and 9.4 µA/ppb) for Cr(VI) for rGO/Me/SnO_2_).	CV	0.07 ppb Cd(II) 0.04 ppb Cr(VI)	[74]
Pyridine functionalized AuNPs/3D rGO/GCE	Cr (VI) ions	25–200 µg/L	1.01 × 10^−2^ (μA/(μg/L)	DPAdSV	1.16 µg/L	[69]
Two-dimensional biphenol-biphenoquinone nanoribbons/silver nanoparticles (AgNPs-BP-BPQ NRs)	Cr (VI) ions	52–5200 µg/L	-	DPV	2.0 × 10^−12^ M	[79]
g-C_3_N_4_/AgM/Nf/GCE (g-C_3_N_4_: graphene carbon nitride; AgM: silver molybdate; Nf: Nafion)	Cr (VI) ions	0.1–0.7 µM	65.8 µAµM^−1^cm^−2^	Amperometry	0.0016 µM	[77]
Au-NPs/MWCNT/chitosan	Cr (VI) ions	-	Quantification limit of 0.02 µg L^−1^	DPV	0.007 μg L^−1^	[66]
A carboxylic amide compound containing pyrrole and pyrene groups	Cr(VI) ions	-	-	Fluorescence	0.106 µM.	[78]
Multi-walled carbon nanotube-neutral red-gold nanoparticles (MWCNTs-NR-AuNPs) modified commercially available screen-printed carbon electrode (SPCE).	Cr(VI) and V(V) ions	Cr(VI) 0.4–80 µM V(V) 3–200 µM	Cr(VI): 0.5137 µA/µM V(V): 0.0688 µA/µM	LSV	Cr(VI): 0.025 µM V(V): 0.42 µM (S/N = 3)	[75]
Screen printed electrodes, Thymine -GO- Carbohydrazide	Cr(VI) and Hg(II) ions	for Hg(II) and Cr(VI) in above 5 ppb	-	SWV	Hg(II) and Cr(VI) were estimated to be one ppb and 20 ppb, respectively.	[24]
GCE/PZrS nanocomposite	Cr(VI) ions	0.55–39.5 µmol/L	-	DPV	64.3 nmol L^−1^	[57]
Ni-Co manganate supported on rGO	Bisphenol-A	0.005–7 µM		LSV	2nM	[80]
Au NPS decorated Ti3C2Tx MXenes	Formaldehyde	-	-	Electrical signals-wireless sensor	92 ppb at RT	[22]
(PANI/rGO)	Acetone	1 to 60 ppm	-	-	1 ppm	[81]
SWCNT/SiPc (Silicon (IV) Phthalocyanine)	NH_3_	0.5–50 ppm	-	Chemiresistivity	0.5 ppm 25–80 °C	[30]
SWCNT/SiPc	H_2_	70–1000 ppm	-	Chemiresistivity	70 ppm	[30]

## Data Availability

Data are contained within the article.
